# Level and pattern of human rabies and dog bites in Techiman Municipality in the Middle Belt of Ghana: a six year retrospective records review

**DOI:** 10.11604/pamj.2017.28.281.14218

**Published:** 2017-11-30

**Authors:** Damien Tioyire Punguyire, Anthony Osei-Tutu, Emmanuel Vikpenibe Aleser, Timothy Letsa

**Affiliations:** 1 Techiman Municipal Health Directorate, Ghana Health Service, Ghana; 2 Veterinary Services Department, Techiman, Ghana; 3 Milimed Hospital, Techiman, Ghana; 4 Volta Regional Health Directorate, Ghana Health Service, Ghana

**Keywords:** Rabies, post-exposure vaccination, pattern, techiman, ghana, dog bite

## Abstract

**Introduction:**

Rabies is a viral zoonotic disease that is transmitted primarily by bites from rabid dogs and has the highest case fatality rate of most infectious diseases in humans. We described a 6-year trend of rabies and dog bites in a peri-urban district in Ghana.

**Methods:**

A record review was conducted in the health facilities in Techiman to identify all human rabies and dog bite cases reported from January 2011 to December 2016. Rabies and dog bite data were extracted from health facilities records. Vaccination status of implicated dogs was extracted from the veterinary records at the Techiman Disease Investigation Farm. Data were summarized using proportions and presented using tables, charts and figures.

**Results:**

Thirteen (13) cases of human rabies were recorded from 2011 to 2016. Complete data was available for 10 cases. Median age of rabies victims was 30 (range 3-80 years). A majority were males (8 representing 61.5%). Eight cases came from rural farming communities, 8 had a previous history of dog bite ranging from two weeks to five months before the onset of rabies symptoms and one reported with non-bite rabies. Case fatality was 100%. A total of 680 dog bites were reported by health facilities. About 50.3% (342) of the victims were males, a majority of bites (47.9%) occurred among children aged 1-15 years. Positive rabies cases among offending dogs ranged from 3.3% in 2016 to 17.6% in 2014.

**Conclusion:**

Mass vaccination of dogs and provision of post-exposure vaccination are needed to reduce rabies transmission.

## Introduction

Rabies is a zoonotic disease caused by Lyssavirus from the family *Rhabdoviridae*. Domestic and wild animals are mostly vulnerable to rabies infection, but the disease is transmitted to humans through bites or scratches from these animals [1]. The rabies virus infects the central nervous system, causing disease in the brain which eventually leads to death. Among unvaccinated population rabies is almost always fatal if post-exposure prophylaxis is not administered before the onset of symptoms [2, 3]. Globally mortality resulting from rabies is estimated between 40,000-70,000 deaths annually, with nearly all deaths occurring in developing countries [4]. These potentially preventable deaths, occur in Africa and Asia where animal control, vaccination programs and post exposure prophylaxis are not universal [1]. The domestic dog is an important vector in the transmission of human rabies, contributing about 97% of all rabies related deaths in humans worldwide [5, 6]. The disease occurs commonly in rural farming communities where measures to prevent dog to human transmission have been poorly implemented [7]. Wound cleansing within a few hours after bites from suspected rabid animal can prevent the onset of rabies and death. Every year, about 15 million people worldwide receive post exposure preventive regimen to prevent rabies at an annual cost of $58 3.5m but most beneficiaries live in developed countries [8]. For many decades now, rabies has been occurring among human population in Ghana. Between 2000 and 2004 alone, there were about 123 clinically confirmed human rabies cases reported by public health facilities across the country [9]. In 2009, an outbreak of rabies in dog and human population in three communities in Bongo District of Upper East region and one border community in Burkina claimed nine lives [10]. However, human rabies cases in Ghana are generally under reported because of weak surveillance system, poor laboratory support and unhealthy socio-cultural factors. Secondly, while majority of human rabies cases are caused by exposure to dog bites [6], information on these bites is limited in the country, making it difficult for effective public health planning in addressing the menace of human rabies in the country. Hospital records revealed that between 2009 and 2012 there were 546 reported cases of dog bites and five cases of rabies in Techiman Municipality [11]. With ever increasing population density and the need for domestic dogs for security and economic reasons in this peri-urban municipality, dog bites and human rabies cases are believed to be on the increase. The need for proper documentation of this trend to inform public health actions on the disease in this geographic area is urgent. We conducted a six year retrospective records review of dog bites and rabies cases reported at health facilities from 2011-2016 to document the trend and the management of these cases in Techiman municipality.

**Profile of techiman:** Techiman municipal assembly is located in the savanna transitional ecological zone in the middle belt of Ghana. It is one of the 27 administrative districts in Brong Ahafo region of the country. The municipality is situated in the central part of the region and lies between longitude 1049'and 2030' west and latitude 8000' north and 7035' south and has annual rainfall between 1,260mm to 1,660mm. It is boarded to the north by Wenchi and Techiman north districts, to the south by Sunyani municipality and Offinso north district, to the west by Tain district and to the east by Nkoranza south [12]. It has a total land surface area of 669.7sqkm with a climate and vegetation that promotes agricultural activities. According to the 2010 population and housing census the municipality has an estimated population of about 170, 000 representing about 6.4% of the region's total population and the highest population density of 318 persons per square kilometer in the region. Close to 65% of the population live in urban areas with the remaining located in rural farming communities. The municipality has 131 communities with a diverse ethnic groups that includes the Akans, Ewes, Dagaabas, Dagombas, Mumprusis, Frafras and almost all tribes in Ghana. Health delivery within the municipality is being conducted by 4 health centers, 9 functional CHPS compounds, 4 private hospitals and clinics, 5 maternity homes and 2 mission hospitals. In particular, the Holy family hospital, owned by the Roman Catholic Church serves as both primary and secondary referral hospital for most health facilities located in the eastern corridor of Brong Ahafo Region.

## Methods

The investigation was carried out from 11^th^ January, 2017 to 23^rd^ March, 2017. It involved review of medical records and interview with health providers within the municipality. We conducted a six year retrospective records review in the Holy family Hospital, the only referral hospital for serious medical conditions, to identify all clinically diagnosed rabies cases during the period. We reviewed the admission and discharge books at the medical and emergency departments of the hospital to identify records of all cases of rabies reported during the period under review. Using the folder numbers, we retrieved and extracted demographic and clinical data of confirmed rabies cases admitted at the hospital. This was complemented by reviewing the District Health Information Management System (DHIMS) which is an internet based platform for storing all data from health facilities in the municipality. We identified annual reported cases of rabies in the municipality. To understand the distribution of suspected rabies dog bites in the municipality, we also reviewed records of all dog bites from all the health facilities in the municipality over the same period of time and how they were managed. We reviewed the rabies status of implicated dogs from the veterinary records at the Techiman Disease Investigation Farm where most dog bites from the health facilities were reported for investigation. We interviewed health professionals on their experience with dog bites; looked for availability of protocols for management of dog bites; assessed the availability of anti-rabies vaccines at the health facilities, source of vaccines and challenges on the management of suspected rabies bites. A contact was made with regional pharmacist to understand the supply chain issues with anti-rabies vaccine. Rabies is clinically diagnosed when person who is suspected to have had a contact with a rabid animal develops one or more of the following: headache, neck pain, nausea, fever, fear of water, anxiety, agitation, abnormal tingling sensations or pain at the wound site. A suspected case of rabies is confirmed in laboratory by detection of viral antigens using direct florescent antibody test of brain tissue. This is not routinely done in the study country. For the purpose of this study, we defined human rabies as any case that a clinician diagnosed as such. A bite was defined as suspected if the dog bit a human without any provocation, if the dog was wild or not vaccinated and if it died or escaped after the bite [13]. An animal was confirmed as having rabies if the Sellar stains on the brain tissues showed the presence of nigre bodies at Techiman disease investigation Farm or if a suspected rabies dog could not be traced for investigation. Data was analyzed in excel and summarized as proportions and presented in tables and figures (graphs).

## Results

**Figure 1:**
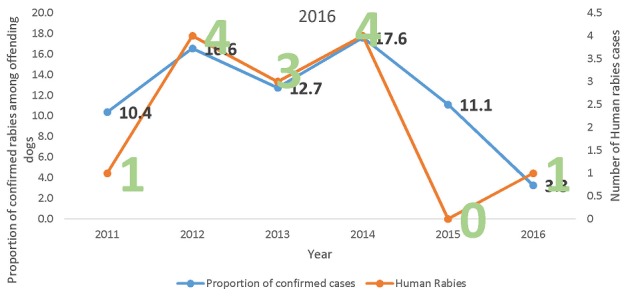
Number of human rabies cases verses incidence of rabies among offending dogs in Techiman Municiparity; 2011-2016

We interviewed 13 health and Agricultural workers comprising 3 medical doctors, 2 physician assistants, 2 nurses, 3 pharmacists, 1 veterinary doctor and 2 technical officers in disease control. Median age of staff was 46 years (minimum 34 and maximum 58 years) and median years of experience 17 (minimum 4 and maximum 28 years). Nine health facilities were visited. In all, 13 cases of human rabies were recorded at the Holy Family Hospital from 2011 to 2016. Demographic data was available for only ten of the cases. Median age of rabies victims was 30 (range 3-80 years). Majority were males (6 representing 60%). Of the 10 cases for which data was available, 8 came from rural farming communities. No two cases came from the same community. Most of the cases were recorded in 2012 (4 cases) and 2014 (4 cases). 8 of the cases had a previous history of dog bite ranging from two weeks to five months before onset of rabies symptoms. Only one reported with non-bite rabies (consumption of rabies dog) and history of exposure was unavailable for 1. Case fatality was 100%. Of the 8 that had history of dog bites, 6 were domestic bites while 2 were stray dog bites. No information was available on the vaccination/or rabid status of the dogs. Table 1 shows a trend in incidence of suspected rabies dog bites investigated by the veterinary department of Techiman from 2011-2016. These dogs had bitten people and were referred by clinicians to the Animal Investigation Farm (Veterinary department) for advice. Confirmed rabies cases among dogs that bit people in the municipality range from 3.3% in 3016 to as high as 17.6% in 2014. The year 2012 recorded the highest number of suspected rabies dog bites of 100/100,000 population and the lowest bites were recorded in 2015, registering 52.6 bites/100,000 population (Table 1). Table 2 shows age and sex distribution of suspected rabies dog bites victims investigated by the Animal Investigation Farm at the veterinary unit in Techiman municipality from 2011-2016. In all, 680 suspected dog bites were investigated by the department. Dog bites were more common among males (342) than females (338). The highest number of dog bites occurred among children aged 5-14 years, recording 321 bites. Among those who reported at health facilities majority (64.7%) were from urban areas. Figure 1 shows annual number of human rabies cases against the proportion of confirmed rabies among dog bites in Techiman municipality from 2010-2016. As proportion of confirmed positive dog bites increased the number of human rabies cases also increased (2012 & 2014) (Figure 1).

**Table 1:** Trend of rabies and dog bites in Techiman, 2011-2016

Year	Dog bites	Bites/100,000	Number & (%) positive cases in dogs	Stray dogs not available for Testing/observation	Human rabies cases	Incidence of Human rabies/100,000
2011	106	65.4	11 (10.4)	14 (13.2)	1	0.6
2012	164	100	26 (15.9)	31 (18.9)	4	2.4
2013	102	61.8	13 (12.7)	23 (22.5)	3	1.8
2014	108	64.9	19 (17.6)	14 (12.7)	4	2.4
2015	90	53.6	10 (11.1)	8 (8.9)	0	0
2016	92	54.1	3 (3.3)	11 (12.0)	2	0.6

The highest dog bites/100,000 occurred in 2012. Most cases of rabies also occurred in 2012 and 2014

**Table 2:** Age/sex distribution of suspected rabies dog bite victims in Techiman from 2011-2016

Age Group in years	Sex		Total
	Male	Female	
Under 1	-	-	-
1-4	65	30	95
5-14	135	96	231
15-44	90	122	212
45-59	25	52	77
Above 60	10	20	30
Unknown	17	18	35
Total	342	338	680

**Figure 2:**
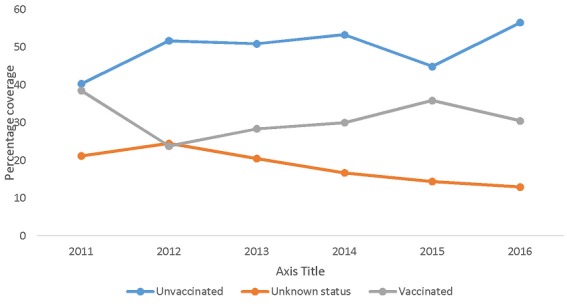
Vaccination status of suspected rabies dog bites in Techiman from 2011-2016

**Figure 3:**
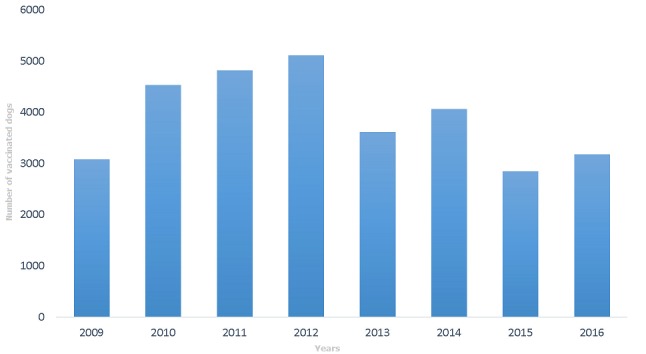
Number of vaccinated dogs per year among the dog population in Techiman Municiparity, 2009-2016

Figure 2 shows the vaccination status of suspected rabies dog bites in Techiman. Less than 35% of the suspected rabies dogs that bit people over the period were vaccinated. About 20% of the offending dogs had unknown vaccination status. Those dogs whose vaccination status was unknown were mostly stray dogs and may not have been vaccinated (Figure 2). Figure 3 shows the trend of vaccination among dog population in the municipality 2009 to 2016. Although, the dog population within the municipality is unknown, the number of vaccinated dogs is believed to be less than 25%. The highest number of vaccinated dog population was in 2012 while the lowest number was in 2015. The number of vaccinated dogs has declined since 2012. Veterinary officers claimed that several attempts to organize mass vaccination campaigns often received low patronage as it is not mandatory for animal owners to have them vaccinated (Figure 3). Of the 9 health facilities visited, 6 referred patients with suspected rabies animal bites to veterinary department for advice. None of the health facilities visited had displayed a protocol for managing dog bites at service delivery points. Majority of clinicians in hospitals around town referred all suspected rabies dog bites to the veterinary department for investigation and advice. Few will ascertain the vaccination status of the dog before deciding what to do. On the other hand, the health centers in the rural areas do not refer dog bites to the veterinary department. Their decision to give post exposure vaccination would depend on the characteristics of the offending dog. Most bites with obvious wounds were cleansed and antibiotics and tetanus anti-serum given. No information was available on whether suspected rabies bite victims received post-exposure vaccination as this information was not documented in the patients' records. No case based forms were filled for human rabies cases or suspected rabies dog bites at the disease control unit of the Municipal Health Directorate. Officers claimed that rabies and dog bites cases are not routinely investigated. Of the health facilities that managed dog bites, only one had post-exposure vaccine available at the pharmacy department. The vaccine was procured from pharmaceutical companies at the open markets and sold to patients. All other health facilities prescribed vaccine for patients to buy from pharmacy shops in town. This is because the regional medical store which supplied health facilities with these important vaccines had not received anti-rabies vaccine since the central medical stores was gutted by fire in early 2015 (Regional pharmacist). None of the pharmacy shops (4) that sold vaccines had temperature monitoring charts in the vaccine fridges. Vaccines were often sold to patients packaged in envelops instead of cold chain boxes.

## Discussion

Our study recorded 13 cases of human rabies diagnosed clinically in Techiman municipality over the period with 100% case fatality. We believe that these numbers may actually represent a gross under-reporting of the true magnitude of the problem in our study area. Rabies commonly manifest clinically with symptoms of mental illness. Anecdotal observations show that the first point of care for people with neuro-psychiatric conditions in our study area is herbalists and spiritual healers where data is not routinely captured by the health system. It could partly be because some cases that presented at the health facilities could have been mis-diagnosed as meningitis or encephalitis while many others could have died in the communities [5]. However, our findings are consistent with other findings in parts of Africa, highlighting rabies as a serious public health problem [5, 6, 14] that requires attention. Most of the deaths from our study came from rural communities. Also, Majority involved male victims. This is consistent with other studies in Africa where human fatalities occur mostly among individuals in rural farming communities with limited access to resources for proper health care [6]. Men are commonly affected by rabies because they often work outdoors during farming and hunting expeditions and therefore come into contact with strayed dogs compared to women who often work indoor in rural areas. Children less than 15 years had the highest exposure to suspected rabies dog bites. This could be due to the fact that children by nature are more likely to provoke dogs, yet they cannot defend themselves. This finding is consistent with studies carried out in Tanzania and Ethiopia [15, 16]. However, another study in New York found no significant difference among sex or age distribution and rabies bite exposure [17], but is in a setting with different pet control program.

In this study, 8 of the 10 cases with information had been bitten by dogs prior to presentation, 1 had handled or eaten a rabid dog and for 1 no history was given. Similar observations were found in other studies where domestic dog bites were responsible for over 94% of human rabies cases [18-20]. Transmission of rabies from non-bite source is rare but it is believed to occur when saliva or brain tissue gets into the eyes, nose, mouth or wounds from scratches, aberrations or open wounds of individuals [21]. In our case, the person was involved in carrying and preparing of meat of a dog that had displayed symptoms of rabies before death and might have been contaminated with the virus through any of the above routes. Of the suspected rabies dog bites that were referred to the veterinary department for investigation, between 3.3-17.6% tested positive for rabies. This result is far higher than the incidence of rabies among dog bites reported in Ethiopia [22]. Moreover, the test used to diagnose rabies in our study was the microscopic examination by Seller's stain test (SST) for Negri bodies. However, this test has low sensitivity when compared with direct fluorescent antibody test (dFAT) [23, 24] implying that the result could have under-estimated the real situation. This poses a major risk of continuous transmission of the disease to human population in the municipality. The situation has also brought to the fore, the challenges facing rabies control in the country and calls for urgent laboratory support in the fight against transmission of rabies. In our study, incidence of rabies among dogs correlates positively with incidence among humans. Because of the role that domestic dogs play in the transmission of rabies to humans, regular anti-rabies vaccination of dogs could help control transmission of the disease to humans. Less than 35% of the offending dogs were vaccinated. This level of coverage is not adequate to prevent transmission of rabies. Vaccination of dogs is one single most important intervention used to control rabies [25]. For example, routine vaccination of dogs was used to eliminate canine rabies in Western Europe and North America [26, 27]. Annual vaccination coverage of 70% in domestic dogs is recommended for effective control of the disease [28, 29].

In our study area, routine vaccination campaigns are organized annually for pet owners but patronage has been poor. This is because Ghana currently does not have an enforceable policy on pet vaccination and coupled with low awareness of the dangers of rabies among the populace makes it unattractive for pet owners to have them vaccinated. We found in our study that only 1 out of the 9 health facilities that were visited during the study period had stockpile of post-exposure vaccines for rabies. The rest would prescribe treatment to be purchased at a pharmacy shop in town. The shops outside hospital setting do not have temperature monitoring charts for medicines. Coupled with frequent interruption of electricity supply in the country and no backup generators for pharmacy shops, could undermine the potency of the vaccines. Vaccines and anti-sera for major conditions such as dog and snake bites are procured and distributed to victims by the Ministry of Health of Ghana at no cost. However, the fire outbreak that gutted down the Central Medical store in 2015 disrupted supply of these essential vaccines [30]. This has forced health facilities to procure such vaccines from other sources, sometimes with doubtful efficacy. Addressing these important supply chain issues is critical for effective management of dog bites in the country. Strangely, none of the health facilities had protocols for managing suspected rabies bites and there was no documentation of patients receiving post-exposure vaccination. Post-exposure guide for the treatment of suspected rabies bite recommends immediate local treatment of wound (categories II and III) and initiation of vaccination at days 0, 3, 7, 14 and 30 while the suspected animal is immediately killed using humane methods and tissues examined using appropriate laboratory techniques or observed if the animal is vaccinated [31]. Treatment may be discontinued if the killed animal tested negative for rabies or if a living animal remains healthy after 10 days of observation [31]. The practice where medical personnel based their treatment decision on the vaccination status of attacks dogs must be discouraged. This is because they are not in the best position to confirm true vaccination status by merely examining the vaccination certificate, particularly, in an environment where one certificate is used to cover more than one dog. Collaboration between health personnel and veterinary workers is required for effective management of dog bites. Finally, we noticed that the existing integrated disease surveillance and response system in the municipality does not document cases of human rabies and dog bites on a timely manner. As a result, these cases are not routinely investigated unlike measles, Yellow fever and Acute Flaccid Paralysis (AFP) cases. As a disease with high case fatality, weak surveillance on rabies could lead to many preventable deaths.

**Study Limitations:** This study is subject to number of limitations. First of all, cases of human rabies and dog bites are based on health facility records which are limited by incomplete records and under reporting. Also, not all cases of rabies and dog bites seek medical care at the orthodox health facilities. This would suggest that our finding could under report the true situation on the ground. Secondly, due to cultural reasons, autopsy is not routinely done in the study area. Suspected rabies deaths were therefore not confirmed by laboratory studies. The possibility of misclassifying rabies deaths in our study cannot be ruled out. Moreover, the study did not assess clinicians' knowledge of rabies case definition. Since diagnosis was based on clinical judgment, assessing clinicians' knowledge on rabies case definition would have been useful in evaluating the validity of the diagnosed rabies cases. However, all suspected cases of rabies were referred to the Municipal hospital which has clinicians with many years working experience. We anticipate their clinical judgment to be accurate. Last but not least, though the trend of dog vaccination was provided, we could not calculate the coverage of dog vaccination since the dog population in the municipality is unknown. Gross numbers alone do not provide any useful information on herd immunity for dogs. Household survey in the study area will be useful in ascertaining vaccination coverage of dogs

## Conclusion

Mortality from rabies is a problem in Techiman. Majority of rabies infections are caused by bites from domestic dogs. Incidence of rabies among dog population in the municipality is high and there is low coverage of vaccination. Rabies is a vaccine-preventable disease. Immunization of all pets (dogs, cats and monkeys) at age 3-4 months and repeating at 1 year, then every year is most effective way of preventing the disease transmission. Therefore a policy requiring mandatory vaccination of domestic animals could improve coverage of rabies vaccination in the country. Victims of pet bites are advised to report to the hospitals for post exposure prophylaxis. Collaboration of all multidisciplinary stakeholders in health and agriculture to ensure supply of post-exposure vaccination could reduce the risk of transmission of the disease. Finally, there is the need implement dog bites surveillance system and strengthen rabies surveillance in the context of the existing integrated disease surveillance and response system (IDSR) to improve early response to the disease.

### What is known about this topic

- Rabies in human population is very common in Ghana according health records;
- The commonest mode of transmission of rabies is through dog bites;
- Records review of rabies cases has been done at tertiary hospital in a metropolitan area of Ghana.

### What this study adds

- The incidence of rabies among offending dogs in this study ranged from 3.3-17.6% implying that rabies is common among dogs in the study area;
- More than 50% of suspected rabid dog bites in the municipality were not vaccinated;
- Health facilities in the study districts are not well equipped with vaccines to provide post-exposure vaccination. The findings have serious implications for the control of rabies transmission to humans. Unvaccinated dogs will transmit the virus to humans through bites who may not be well managed enough to prevent the development of clinical rabies. Mass vaccination of dogs and regular supply of effective post-exposure vaccines are needed to reduce transmission to humans.

## Competing interests

The authors declare no competing interest.
